# Learning global health: a pilot study of an online collaborative intercultural peer group activity involving medical students in Australia and Indonesia

**DOI:** 10.1186/s12909-016-0851-6

**Published:** 2017-01-13

**Authors:** Mark Ambrose, Linda Murray, Nicholas E. Handoyo, Deif Tunggal, Nick Cooling

**Affiliations:** 1 School of Medicine, University of Tasmania, Private Bag 34, Hobart, 7000 Australia; 2 Medical Faculty, University of Nusa Cendana, Adi Sucipto St. Penfui, Kota Kupang, 85000 Timor Island, NTT Province Indonesia

**Keywords:** Global health, Tropical disease, Peer learning, *e*-learning, Intercultural learning, Internationalisation

## Abstract

**Background:**

There is limited research to inform effective pedagogies for teaching global health to undergraduate medical students. Theoretically, using a combination of teaching pedagogies typically used in ‘international classrooms’ may prove to be an effective way of learning global health. This pilot study aimed to explore the experiences of medical students in Australia and Indonesia who participated in a reciprocal intercultural participatory peer *e*-learning activity (RIPPLE) in global health.

**Methods:**

Seventy-one third year medical students (49 from Australia and 22 from Indonesia) from the University of Tasmania (Australia) and the University of Nusa Cendana (Indonesia) participated in the RIPPLE activity. Participants were randomly distributed into 11 intercultural ‘virtual’ groups. The groups collaborated online over two weeks to study a global health topic of their choice, and each group produced a structured research abstract. Pre— and post-RIPPLE questionnaires were used to capture students’ experiences of the activity. Descriptive quantitative data were analysed with Microsoft Excel and qualitative data were thematically analysed.

**Results:**

Students’ motivation to volunteer for this activity included: curiosity about the innovative approach to learning; wanting to expand knowledge of global health; hoping to build personal and professional relationships; and a desire to be part of an intercultural experience. Afer completing the RIPPLE program, participants reported on global health knowledge acquisition, the development of peer relationships, and insight into another culture. Barriers to achieving the learning outcomes associated with RIPPLE included problems with establishing consistent online communication, and effectively managing time to simultaneously complete RIPPLE and other curricula activities.

**Conclusions:**

Medical students from both countries found benefits in working together in small virtual groups to complement existing teaching in global health. However, our pilot study demonstrated that while intercultural collaborative peer learning activities like RIPPLE are feasible, they require robust logistical support and an awareness of the need to manage curriculum alignment in ways that facilitate more effective student engagement.

**Electronic supplementary material:**

The online version of this article (doi:10.1186/s12909-016-0851-6) contains supplementary material, which is available to authorized users.

## Background

It is widely recognised that for medical doctors to practice in the globalised and interdependent world of the 21^st^ century, they must have a broad content knowledge of current and emerging global health issues. Since the call for additional teaching on global health in medical schools over a decade ago, the focus has been on improving content knowledge about global influences on human health (such as economic, environmental and social determinants) and infectious disease [1, 2]. However, the development of intercultural skills that enable doctors to effectively communicate with and appropriately treat patients of diverse cultural backgrounds are also crucial [1, 3–5].

One way medical schools in North America, Europe, and Australia [6–8] have begun to prepare students for the impact of globalisation is through the so called “internationalisation” of their curricula. While there are different interpretations of the concept of an internationalised medical curriculum, a recent review suggested it should incorporate at least three core elements: teaching on global health, programs that foster student mobility, and learning activities that facilitate the development of intercultural competencies [8]. Here, intercultural competence is understood as “the ability to interact effectively and appropriately in intercultural situations, based on specific attitudes, intercultural knowledge, skills and reflection” [9, 10]. Models for internationalising the medical curriculum range from minor add-on programs through to more integrated approaches that promote transformative learning [7, 8]. Global health content is generally taught in traditional formats such as lectures, tutorials and workshops [11]. Many universities also offer international field experiences but not all students can afford to participate in these programs and they can expose students to health and personal safety risks [12].

A current challenge in internationalising our medical curriculum lies in developing pedagogies to effectively teach the central themes of global health and cultural competence [8]. The learning methodology itself, and not just subject content, may be critical to enhancing students’ understanding of these themes. It is likely that global health could be more effectively learnt in medical schools through more innovative teaching and learning techniques, and through engaging with the cultural diversity of staff and students already present in the classroom—the so-called ‘internationalisation at home’ paradigm [13]. This would require medical educators to aquire an understanding about the type(s) of learning approaches that facilitate developing knowledge about global health, as well as the skills and attitudes required for cultural competence [8].

A review of the medical education literature reveals very few studies on teaching and learning strategies that can be used to facilitate medical students’ understanding of global health issues. Two frequently cited qualitative studies by Godkin and Savageau [14] and Niemantsverdriet and colleagues [15] reported that medical students involved in international clinical environments believed that they had developed appropriate clinical skills and positive attitudes about working with multicultural populations. However, these studies did not make clear what teaching and learning processes fostered the development of such positive outcomes.

With few examples in practice to draw on, educational theory may help predict what pedagogies are likely to enhance the learning of global health in medical schools [16, 17]. Contemporary learning pedagogies such as *e*-learning appear to be theoretically useful for the collaborative and inter -connected learning that students may need in global health [18]. Online learning has been shown in one recent study to be attractive to students studying global health because of its perceieved interactivity, and multi-media approach [19]. Against this background then, it ought to be possible to design online approaches for learning global health that would allow students to teach and learn from each other.

In this context, peer learning is underpinned by Vygotsky’s social constructivist educational theories [20] that suggest that student peers working together on an authentic assessment task will build their knowledge about the task through both informal and formal interaction(s) [21, 22]. Peer learning is assumed to be associated with deep rather than surface approaches to learning, and hence more likely to develop in students important higher learning skills such as critical thinking, intellectual curiosity, problem-solving, logical and independent thought, communication and information management skills, intellectual rigor, creativity and imagination, ethical practice, integrity and tolerance [21, 23].

There are different forms of peer learning, including peer tutoring, peer teaching, and reciprocal peer learning [24]. Peer tutoring and peer teaching, where one student learns from interacting with a more experienced colleague, are more commonly used in medical schools and appear to dominate the scholarly literature in medical education [21, 25, 26]. In contrast, the educational value of reciprocal peer learning, where students can act as both teacher and learner, and where the power in student relationships is considered to be equal, has perhaps been underused and less well studied in medical education [21, 22]. Reciprocal peer learning is suggested to be a powerful strategy for learning [27], and hence may well be effective at facilitating students’ learning about global health. Furthermore, it has been argued by several authors that creating opportunities for students to engage interculturally on peer collaborative projects focussed on global health, and thereby potentially exposing them to culturally diverse opinions and perspectives, including discussions on varying healthcare delivery systems, can lead to a more comprehensive understanding of global health issues [28–30]. More importantly perhaps for present purposes, the availability of increasingly sophisticated and user friendly online web 2.0 tools for document creation/ sharing and communication means that opportunities for intercultural peer collaborative learning are no longer limited to face to face activities [31].

The two medical schools participating in this research study were from different cultural contexts (Australia and Indonesia), and have been collaborating since 2010. To increase the opportunities for students from both medical schools to experience cultural exchange and learn global health within a group of international peers, and without the need to travel, an innovative learning activity was developed. This activity involved a *reciprocal intercultural, participatory, peer e*-*learning* (RIPPLE) approach with third year medical students from the University of Tasmania (UTAS, Australia) and the University of Nusa Cendana (UNDANA, Indonesia). Our research aimed to explore the experiences of medical students from UTAS and UNDANA who participated in RIPPLE to determine how this pedagogy facilitated their learning about global health.

## Methods

### Setting and participants

The study took place at the medical schools of UTAS Hobart campus in Tasmania, Australia, and at the UNDANA Kupang campus in NTT, Indonesia. Communication between the participants at the two schools occurred online and via video-conference. Study participants were volunteer third year undergraduate medical students from these two institutions. The UNDANA cohort consisted of 100% Indonesian nationals mostly from the NTT Province, while the UTAS cohort comprised of 80% domestic students mostly born in Australia and 20% of full fee paying international students, mostly from Singapore and Malaysia.

The UTAS medical course is a five year undergraduate program with an emphasis on case based learning (CBL) and the UNDANA course is a 11 semester five-and-a-half year undergraduate program that is aligned to a more didactic national curriculum. At the time of participating in RIPPLE UTAS students were engaged in a two-week module on global health, which occurred alongside various clinical rotations and teaching on the neurological system. In contrast, at UNDANA, RIPPLE was delivered during their semester five when the teaching focus was on the urogenital, reproductive and gastrointestinal systems, and ethics and law, with no teaching on global health.

**Fig. 1:**
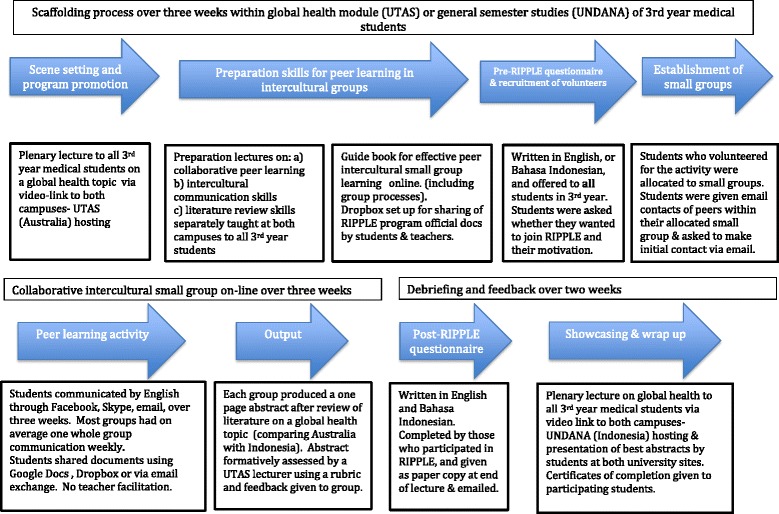
RIPPLE program components

The elements of the RIPPLE program are shown in Fig. 1, including the preparation and debriefing phases.

The voluntary RIPPLE program was marketed to third year medical students at both universities through a project information package provided to each student via e-mail, and through classroom announcements explaining the purpose of the progam.

All third year students (at UTAS and UNDANA) regardless of their interest in RIPPLE were asked to complete a pre-RIPPLE activity questionnaire (Additional file 1), which included an invitation to participate in RIPPLE. Students who agreed to participate in RIPPLE were also invited to complete an additional post-RIPPLE activity questionnaire (Additional file 2).

### Educational activity

The design of the small group process was based on recomendations from previoulsy successful on-line small group collaborative projects [31, 32] (see Table 1). Students who volunteered for RIPPLE were randomly allocated to 11 virtual small groups comprised of up to eight students with at least two students from each university per group. Students were instructed on how to appropriately connect, communicate, and work with each other in an online environment using a variety of online communication tools (eg. Facebook, Skype, Webchat 2.0, and e-mail); how to share documents (Dropbox, Google docs); and how to work interculturally through a 60 min lecture and guide booklet. It was the responsibility of each peer group to determine their preferred web tools. RIPPLE was run over a three week period, alongside the usual medical course activities at the respective medical schools, and students were not supervised during their online RIPPLE collaborations. A lecturer at both medical schools was available to answer students’ questions by email, however. Dedicated in-class time was not provided, so participants mostly collaborated in their small groups after formal course hours. There is a three hour time difference between Kupang and Hobart.

**Table 1:** Measures to increase effectiveness of learning groups

	Creating the groups	Structuring learning activities	Facilitating group interactions
Important factors for creating effective face to face and virtual learning groups [32]	Group size Group heterogeneity	Positive interdependence Accountability	Developing cooperative group skills Developing group norms
Methods for promoting effective learning groups in the RIPPLE activity	Ensured small groups (up to eight students) and mixture of UTAS & UNDANA students (min two per group) and genders	Guide book Students followed up by local academic to ensure tasks (generation of one page abstract & questionnaires) was completed on time	Guide book Lecture on working in collaborative small groups Email support from academics at both universities to troubleshoot difficulties

Each virtual group chose a topic from a list of potential research topics relevant to global health and tropical disease (Additional file 3). Most topic tasks asked for a comparison of the situation in Australia and Indonesia. Groups were required to produce a 500 word structured literature review in an abstract format, which introduced their research topic, outlined the search strategy used to research the relevant project, and summarised key research findings. All groups were provided with a criterion referenced assessment (CRA, or marking rubric) detailing expectations for the research abstract.

### Data collection instruments

#### Pre-RIPPLE questionnaire

A pre-RIPPLE activity questionnaire was distributed to all third year medical students at both medical schools before they decided whether or not to participate in the RIPPLE program. The questionnaire included yes/no response questions about their intention to participate and two open-ended questions asking students to list any factor(s) that motivated them to volunteer to take part in RIPPLE, as well as their expectations of RIPPLE. If students did opt to volunteer for this small group activity they were asked to indicate past experiences with peer and intercultural peer learning, as well as experiences working in online environments (Additional file 1).

#### Post-RIPPLE questionnaire

A post-RIPPLE questionnaire was distributed only to those students who volunteered to take part in the RIPPLE program. This questionnaire contained ten likert scales and two open-ended questions asking students to respond to the overall design of the program; experiences with reciprocal intercultural peer learning; and perceptions on the impact of this experience on their overall understanding of global health issues and tropical disease in Australia and Indonesia (Additional file 2).

At both medical schools, the questionnaires were administered in class, and also sent to participating students via e-mail and/or by the relevant university student management systems. The pre-RIPPLE questionnaire (Additional file 1) provided to all third year students was administered after a lecture on a global health topic, and the post-RIPPLE questionnaire (Additional file 2), only provided to participating students, was administered after the concluding program session.

### Quantitative and qualitative analysis

Responses captured on a four-level (strongly disagree, disagree, agree, and strongly agree) likert scale were later collapsed (because of low student numbers) into binary variables (agree/disagree). Questions with answers that were binary (yes/no) were analysed with Microsoft Excel and expressed as percentages. Qualitative thematic analysis was conducted for the written responses to the remaining open-ended questions (two questions in the pre-RIPPLE survey and three questions in the post-RIPPLE survey) by two researchers who independently coded the data to inductively generate themes. A third researcher then reviewed the themes to ensure confirmability [33].

## Results

### Recruitment

At UTAS, 62 students out of 100 students in the third year class completed the ‘pre-RIPPLE questionnaire’, a response rate of 62%. Of those, 49 students (49% of the whole class) volunteered to participate in RIPPLE. In contrast at UNDANA, 24 ‘pre-RIPPLE questionnaires’ were completed from a class of 33, representating a response rate of 73%, and 22 students (67% of the whole class) indicated “yes” to participating.

### Non-participants in RIPPLE program

Forty nine students (UTAS *n* = 38 and UNDANA *n* = 11) who chose not to participate in the RIPPLE program completed the pre-RIPPLE questionnaire (Additional file 1). Most students in this category cited reasons such as “lack of time” and “other work and study commitments” as their reason(s) for non-participation. In addition, some UTAS students cited previous experience(s) with studying global health issues as a reason not to participate believing that they had already acquired intercultural competencies, while others stated they expected to “learn about global health issues and tropical disease in lectures”. Some UNDANA students cited apprehension about their competency in oral English as a barrier to them participating in RIPPLE.

### Pre-RIPPLE survey results

#### Participation in RIPPLE program

In all, there were 49 students from UTAS and 22 from UNDANA who volunteered for the RIPPLE program. The pre-RIPPLE questionnaire results presented in Table 2 reflect only the responses of RIPPLE participants. The results of closed-ended questions revealed that any form of peer learning, including intercultural peer learning, was not a recognised experience for the UNDANA students. The UTAS students also reported on little experience with intercultural peer learning (*n* = 3, 4%), although the majority were familiar with other forms of peer learning opportunities in their medical course (*n* = 49, 79%). The UTAS students also indicated more prior experience with online tools for peer learing (52%) compared to their UNDANA peers (0%) .

**Table 2:** Pre-RIPPLE activity questionnaire responses

Question	University of Tasmania (Australia)				University of Nusa Cendana (Indonesia)			
	Yes N (%)	No N (%)	N/A N (%)	Total N (%)	Yes N (%)	No N (%)	N/A N (%)	Total N (%)
Have you experienced peer learning in any other unit of study at university?	49 (79)	13 (21)	0 (0)	62 (100)	1 (4)	23 (96)	0 (0)	24 (100)
Have you used online tools to engage in peer learning in any other unit of study at university?	32 (52)	17 (27)	13 (21)	62 (100)	0 (0)	24 (100)	0 (0)	24 (100)
Have you experienced intercultural peer learning in any other unit of study at university?	3 (4)	46 (75)	13 (21)	62 (100)	0 (0)	24 (100)	0 (0)	24 (100)

Participants provided open-ended answers to questions on what motivated them to volunteer to take part in RIPPLE, and their expectations from engaging in the program. From their answers four themes emerged.

### Curiosity

Many UTAS students expressed curiosity about the idea of RIPPLE and thought it could generate interesting experiences:

“*I’m not sure what to expect, which is why I signed up*” (UTAS student 3)

“*Exciting opportunity, sounds fun and not too time consuming*” (UTAS student 33)

We found that “curiosity” was not overtly reported by the UNDANA students.

### Knowledge expansion

Most students indicated they expected RIPPLE would expand their knowledge of global health:

“*(I wish to) gain a broader understanding of global health and tropical disease*” (UTAS student 6)

*I wish to “learn from students in Australia”….and “improve skills to work in globalised medicine*” (UNDANA student 8)

“I *want to know all about knowledge, not only the science but also the culture to open my mind, I want to increase my skills so I can help my people …”* (UNDANA student 15)

### Building relationships for professional and personal growth

Equally UTAS and UNDANA participants stated that they expected to gain skills to help them in future employment and expressed hope to form authentic relationships with intercultural peers:

“*Indonesia is close to Malaysia and I want to work there one day*” (UTAS student 23)

“*I can improve my English speaking, I can know more about Australia’s culture, I can improve my knowledge about medicine and share our knowledge in medical study*” (UNDANA student 6)

In fact over half of the Indoenesian students expressed a desire to improve their English skills:

“*I expect to developing my skill and core competencies for medical doctor preparing for practice in a globalised world seerving diverse cultures, get a new friends from overseas and also I expect to have opportunity to have students exchange*” (UNDANA student 12)

“*I have an interest to meet and talk with people. This provides me an opportunity to get to know people and work with them. It is the process of sharing our experiences that makes me enjoy myself*” (UTAS student 13)

### Innovative learning opportunity with global peers

UTAS and UNDANDA participants also reported on their expectations to improve their learning skills and grow their intercultural awareness stating they expected to “learn in ways different to my usual style”; “learn or gain an appreciation for healthcare in a different country”; and “gain awareness that I am part of a global community”.

“*It’s good to be engaged in intercultural peer learning, because we can learn from doctors in Australia, the knowledge that we don’t have here*” (UNDANA student 7)

“*Hope to learn in a way that is different to my usual style*” (UTAS student 4)

“*Interesting way of learning with cross-cultural interaction*” (UTAS student 7)

“*I hope this program will bring benefits for myself, my faculty and my friends from overseas countries*” (UNDANA student 6)

“*Cross-cultural interaction is enriching in that it provides for opportunity to see or appraise ideas from different aspects. It also encourages unity*” (UTAS student 14)

### Post-RIPPLE survey results

At the completion of the RIPPLE, 18 UTAS students and 20 UNDANA students completed the post-RIPPLE questionnaire (Additional file 2), which was an overall response rate of 37 and 90%, respectively. Responses to the post-RIPPLE questions clearly indicated that most UTAS and UNDANA students agreed that the intended learning outcomes of the program were clearly explained, and understood why peer learning was part of the program (See Table 3). Furthermore, most participants agreed that the CRA/marking rubric provided helped them to achieve the intended learning outcomes linked to their group assignment.

**Table 3:** Post survey response

	University of Tasmania			University of Nusa Cendana		
	Agree N(%)	Disagree N(%)	Total N(%)	Agree N(%)	Disagree N(%)	Total N(%)
It was clearly explained why peer learning was part of this program	15 (83)	3 (17)	18 (100)	15 (75)	5 (25)	20 (100)
The intended learning outcomes were clearly outlined	13 (72)	4 (22)	17 (95)	14 (70)	6 (30)	20 (100)
The marking rubric helped me achieve the intended learning outcomes	10 (56)	7 (39)	17 (95)	19 (95)	1 (5)	20 (100)
Online tools made it easy to communicate with our peers from overseas	5 (28)	12 (67)	17 (95)	16 (80)	4 (20)	20 (100)
Working in small groups helped me achieve the intended learning outcomes of the learning activity	7 (39)	11 (62)	18 (100)	17 (85)	2 (10)	19 (95)
The intercultural group work allowed me to apply and deepen my understanding of global health	7 (39)	9 (50)	16 (89)	17 (85)	3 (15)	20 (100)
The intercultural peer project made me aware of similar/different cultural approaches to global health issues	10 (56)	7 (39)	17 (95)	16 (80)	3 (15)	19 (95)
I appreciated learning about global health issues by working with my local and international peers, instead of attending traditional lectures	10 (56)	6 (33)	16 (89)	18 (90)	2 (10)	20 (100)
The project gave me an opportunity to learn how overseas students think about global health issues	5 (28)	12 (67)	17 (95)	18 (90)	2 (10)	20 (100)
The on-campus time allocated to discuss the peer-group project was sufficient	12 (67)	6 (33)	18 (100)	9 (45)	11 (55)	20 (100)

Although the UTAS and UNDANA participants appeared satisfied with the overall design of the intercultural peer learning activity, there was a difference in opinion about whether the use of online tools made it easy for them to communicate and work with each other. Overall, 80% of the UNDANA students agreed that online tools made communication easier, which contrasted with most UTAS students (67%) strongly disagreeing. This trend was consistent with their responses to the question on whether working in small groups helped them achieve the intended learning outcomes (only 39% of UTAS students agreed compared to 85% of UNDANA students), and whether the intercultural group work allowed them to deepen their understanding of global health (39% [UTAS] and 85% [UNDANA], respectively). This difference in responses between the two cohorts extended to how well they percieved they could learn about global health with their overseas peers. While 90% of the UNDANA students agreed that they were able to learn how their overseas peers think about global health issues, most (67%) of the UTAS students strongly disagreed. Students from UNDANA were also less likely to agree that there was sufficient time available to discuss the peer-group project (45% UNDANA vs 67% UTAS).

Three main qualitative themes also emerged from the open-ended questions in the post-activity RIPPLE questionnaire. These were: the benefits of “sharing”, benefits of improved content knowledge, and frustration.

### Benefits of “sharing”

The majority of UTAS and UNDANA participants reported benefits from learning about global health issues from their local and international peers, instead of by content delivery in traditional lecture settings. Students reported that they “understand the challenges facing different countries more,” and many students from UNDANA indicated that the peer-to-peer nature of the program formed part of their learning experience:

*“We can learn and share with foreign students”* (UNDANA student 16)

*“To get global health information, especially in Australia; have foreign friends; to know the different health challenges in Indonesia and Australia and then discuss together”* (UNDANA student 5)

“*Improve English (read, write, listen); learn a different culture; learn the underlying causes of health differences between Indonesia and Australia*” (UNDANA student 2)

### Benefits of improving content knowledge

Interestingly, beside the benefit of the peer-to-peer nature of the RIPPLE program, students from both medical schools indicated that the main benefits they gained from RIPPLE involved learning new content in global health and tropical diseases:

“*Increased knowledge and understanding about a disease related to epidemiology, diagnosis and treatment, and comparison between Indonesia and Australia*” (UNDANA student 17)

*“The introductory lectures…helped me to gain perspective on why there is a need to have a cross-cultural/internationalised learning in this area*” (UTAS student 3)

### Frustration

The theme of “frustration” also emerged in the answers of most students, especially in regards to establishing consistent online communication, time-management, and successfully using technology.

Students from both medical schools described frustration about establishing consistent communication with their group members, which seems to have prevented them from completing the group task and building authentic learning relationships:

*“There were many delays with communication which made the exchange process a bit difficult, however eventually it came together”* (UTAS student 17)

“*It appeared that (some) UNDANA students did not have available internet access. This was exacerbated by reduced internet access for some UTAS students related to them being on rural week*” (UTAS student 26)

The lack of communication also contributed to a lack of motivation about completing the task and thus engaging in group learning:

*“Unfortunately, communication between the members of our group was relatively limited. This resulted in reduced motivation in the more active members of the group.”* (UTAS student 5)

Most of the frustrations regarding communication stemmed from the availability of technology and access to the internet for both student cohorts. This meant that they could not access or reply to communications sent by other members of their group. Limited internet access also affected the timely completion of other aspects of the learning activity such as research:

*“Hard to access journals because they were not free open access journals”* (UNDANA student 7)

Another barrier to collaborative learning was time management:

“*Different time zones between Indonesia and Australia, the busy activities of each student caused not-so effective communication*” (UNDANA Student 1)

*“(hard to) manage time to share with each other because each student has their own tight schedule*” (UNDANA Student 12)

Although apparently a source of frustration, using an online platform also allowed some students at least to gain an appreciation for the difficulties faced by their international peers, thereby engendering mutual understanding and empathy:

*“It has allowed me to understand the various challenges and issues faced in another country”* (UTAS student 11)

Finally, when asked how the peer learning activity could be improved for future student cohorts the majority of UTAS and some UNDANA participants suggested “better regulation of the task”; “more support for groups”; and “better timing in the curriculum”.

## Discussion

The present study attempted to fill a gap in the medical education literature regarding the educational theories that can be used to effectively inform the design of global health programs involving international medical students. It has been argued that medical schools should plan to deliver global health curricula as truly transformative learning experiences [8]. The criteria for the transformative learning model in global health suggested by Murdoch-Eaton et al. [7] includes : *Recognition and utilisation of international staff and students as resources and co- developers of curricular material; utilisation of international students’ experiences to not only contribute to the sessions but also to develop session material sensitive to their needs; accommodation of students’ culturally different learning styles and preferences; inclusion of group tasks where members are from different cultures; and utilisation of web technologies including online networking and liaison with schools and students from international schools to facilitate co-learning*. The RIPPLE activity used in the present study integrated most of these criteria in one learning experience.

In our teaching and research context, we see an ideal opportunity for deeper collaboration between UTAS and UNDANA around how to embed global health, tropical disease, and intercultural aspects of health care into the curricula of the respective medical schools. On reviewing the relevant research literature, however, we note that while there is increasing literature on the development of core components of a global health curriculum for medical schools [1, 34, 35], there appears to be little, if any, research discussing whether teaching institutions (worldwide) engaged in intercultural partnerships have aligned or even shared relevant aspects of their medical curricula in global health.

We found that medical students at an Australian and an Indonesian medical school were enthusiastic about participating in global health learning using the RIPPLE learning approach. The emergent themes suggest that students’ positive attitudes toward participating in the RIPPLE program stemmed in part from their motivations and expectations to enhance their own academic and personal growth, as well as to develop their intercultural awareness. Other studies [14, 15, 29] have similarly reported that undergraduate medical students welcome the opportunity to learn in intercultural environments especially when actively participating in authentic learning activities. The pre-RIPPLE survey response rate for the UNDANA students also seems to suggest that they were more motivated to volunteer to participate in RIPPLE than their UTAS counterparts. While the precise reason(s) for this difference in motivation level are not known, a closer inspection of the UNDANA students’ responses to the pre-RIPPLE activity survey open-ended questions revealed that they were motivated to participate in RIPPLE by factors concerned more so with personal growth than simply wanting to learn about global health issues. Thus for example, the overwhelming majority of the UNDANA students reported on their desire to “develop international friendships” and “improve oral/written English skills”. In addition, the UNDANA students also reported less exposure to global health content, and forms of learning such as peer learning, intercultural peer learning, and online learning compared to their UTAS counterparts (Table 2), perhaps reflective of their local educational mileau [36] and culture [37, 38]. Indonesian students usually encounter didactic teaching [36–38], and hence their motivation to participate in the RIPPLE program might well have stemmed from their desire to experience these “new” forms of learning, which are otherwise more often encountered by their UTAS counterparts (Table 2). It is important to point out here, however, that during the RIPPLE period there were differences in course commitments at the two medical schools, including examinations and general workload all of which may have impacted upon the pre-RIPPLE survey response rates by the UTAS and UNDANA students, as well.

At the completion of the RIPPLE activity, participants reported that they had appreciated learning by working with their local and international peers, and that the project had given them an awareness of global health issues. There is a substantial body of literature documenting the benefits of peer learning, which asserts that peer learning contributes to a deep rather than surface approach to learning [21, 22, 39]. We did not directly measure the extent of deep learning that may have occurred over the course of RIPPLE, although the high quality of peer group generated research abstracts on global health topics provides some indirect evidence for the use of certain of the microskills usually associated with deep learning- synthesis of complex ideas, reflective learning, and working collaboratively to integrate different viewpoints [22]. Moreover, RIPPLE was designed to include recognised promoters for deep learning like formative assessment and feedback [40] and synchronous online discussions with peers, for example [41]. There was a difference in the students’ ‘perceived’ level of deep learning of global health during RIPPLE, however. For example, the UTAS students were less likely to agree that RIPPLE lead to a “deeper learning of global health issues”, a response that seemed to be associated with these students’ frustrations with establishing consistent online communication. The deep learning of global health issues, as well as the acquisition of cultural competence, will be explored more comprehensively in future iterations of RIPPLE, where the aim will be to tease out the impact(s) of potential intercultural differences.

Despite reporting by the UTAS and UNDANA students that they had appreciated learning by working with their local and international peers, and that the project had given them an awareness of global health issues, the execution of RIPPLE was not without its difficulties. In particular, study participants expressed mixed feelings about whether the use of online tools was an effective way to work and communicate with their overseas peers. It has been suggested that the availability of e-mail and the growth of the internet and its associated communication tools should make shared learning between medical students in different parts of the world more possible [2]. Surprisingly, the majority of UTAS participants, the cohort who reported having the greatest exposure to online peer learning, also expressed the greatest dissatisfaction with using online tools. Unlike their UNDANA counterparts, an overwhelming number of UTAS students became frustrated with the *e*-learning logistics, complaining about not being able to (i) establish a consistent internet link; (ii) synchronise a time to communicate online; and (iii) receive emails in a timely fashion, which seems to have left them feeling disenfranchised. Reports from other international online classrooms reflect similar challenges to those experienced during the RIPPLE program — in technology, time and communication [9, 42–44]. However, a major difference in our study is that we had little teacher supervision during the three week’s of RIPPLE. We tended to follow evidence suggesting that students are able to engage in reflection, critical analysis, and debate during online discussions irrespective of whether there is teacher facilitation [45]. Moreover, we were guided by Cheung et al [46] who found that peer norms and self efficacy to use technology rather than media or teacher influences determined the choice of web 2.0 tool use in online discussions amongst peers. Thus, the current RIPPLE design that allowed students to freely communicate with each other and develop their own organisational structure might have conversely bypassed the benefit of direct supervision to navigate through any potential difficulties. This idea is perhaps underscored by the majority of UTAS and some UNDANA participants who reported in the post-RIPPLE survey that RIPPLE could be improved by “better regulation of the task”; “more support for groups”; and “better timing in the curriculum”. In future iterations of RIPPLE, we will aim to provide upskilling in peer learning, *e*-learning and intercultural learning (beyond the brief lecture and RIPPLE guide booklet) prior to student engagement, as well as tutor facilitation during online collaborations. Intriguingly, the UNDANA students who reported having no prior experience with online peer learning activities felt that the online tools used in this study were an effective way to work and communicate with their overseas peers. The reason(s) for these positive attitudes remain unknown but it has been suggested that when using online tools “to some learners the feelings of community and connectedness are more important than to others” [35].

Overall our findings do suggest, however, the need to guide students on the appropriate use of online chat tools, the need to provide structure in online interactions, and the need for better timing of these sessions and embedding into the core curriculum of the two medical schools.

### Limitations

A key limitation of this study was its reliance upon self-reported data, and the low response rate from the UTAS students to the post-RIPPLE survey, which was conducted during their exam period. Moreover, it was a pilot study involving only a small sample of volunteer third year medical students from the two universities. These low response rates mean that our quantitative findings could provide some useful description for our program but cannot be generalised to other contexts. Because students volunteered to participate, this study might have missed capturing other important issues relevant to the reciprocal intercultural peer learning exercise. For example, students holding negative views on peer learning and/or intercultural peer learning might have chosen not to volunteer for the RIPPLE program. In addition, because of the small group work nature of this project, there is a potential for bias in the post-RIPPLE survey reporting. Participants with positive or negative views might well have influenced the opinions of their colleagues in the same peer group, thereby introducing bias. It is likely that the positive responses from many participants could have been affected by the enthusiasm of the teaching staff and the fact there were expectations of success expressed for this pilot study — the so called Hawthorne effect [47]. The socio-cultural differences between UTAS and UNDANA may have also affected student participation. Language barriers between the two medical student cohorts may have resulted in missed information or interpretive differences during RIPPLE, since English is not the official language for the UNDANA medical students.

## Conclusion

The third year medical students at UTAS and UNDANA who participated in the present study recognised the impact of globalisation on their medical training. Importantly, the opportunity for them to engage in intercultural learning in the online environment was enthusiastically welcomed, was technically feasible, and appeared to be appreciated as a useful addition to only lecture-based teaching. The reciprocal peer learning approach enabled students to compare and contrast cultural approaches to global health and tropical disease issues relevant to Australia and Indonesia. Finally, from this study emerged two recommendations for any future reciprocal intercultural peer learning activities, namely: (i) the need to ensure that teaching methodologies are aligned as much as is possible, or at the very least that participating student cohorts are briefed on unfamiliar teaching practices in formal (bridging) training sessions; and (ii) the need to provide appropriately timetabled and supported online learning environments. Educational theory predicted a number of strategies including peer learning that could enhance the learning of global health. However, the results of our study demonstrated that in practice these strategies need significant scaffolding; modification(s) in part at least to suit the local coxtext; and logistical support to achieve effective internationalisation ‘at home’.

## Additional files

Additional file 1: Pre-RIPPLE student survey. (DOCX 72 kb)
Additional file 2: Post-RIPPLE student survey. (DOCX 15 kb)
Additional file 3: List of topics for peer group discussion. (DOCX 16 kb)

## Abbreviations

NTT: Nusa tenggara timur
RIPPLE: Reciprocal, intercultural, participatory peer e-learning activity
UNDANA: University of nusa cendana
UTAS: University of Tasmania
