# Supplementary material for: Learning global health: a pilot study of an online collaborative intercultural peer group activity involving medical students in Australia and Indonesia
Source: BMC Med Educ. 2017 Jan 13;17:10. doi: 10.1186/s12909-016-0851-6 (PMC5237179; doi:10.1186/s12909-016-0851-6)
Supplement: Additional file 1: — Pre-RIPPLE student survey. (DOCX 72 kb) [file 12909_2016_851_MOESM1_ESM.docx]

**Additional file 1. Pre-RIPPLE activity student survey**

***Pre-RIPPLE activity student survey***

**Intercultural Peer *e*-Learning in Global Health**

This short questionnaire is intended to explore the effectiveness of intercultural peer learning for teaching global health content. We anticipate this survey will take around five minutes to complete.

1. Do you intend to participate in the Intercultural Peer *e*-learning Global Health project?

YES NO

**If you have answered NO**:

- 1. Please comment on any factor(s) you believe have impacted your decision NOT to participate in the Intercultural Peer e-learning Global Health project.

- 1. Please list any other activities at the University of Tasmania/UNDANA you are engaged in that are providing an opportunity for you to learn about global health and develop cultural competence?

**If you answered YES:**

- 1. Have you experienced peer learning in any other unit of study at the University of Tasmania/UNDANA?

YES NO

- 1. Have you used online tools to engage in peer learning in any other unit of study at the University of

Tasmania/UNDANA? YES NO

- 1. Have you experienced intercultural peer learning in any other unit of study at the University of

Tasmania/UNDANA? YES NO

Open ended questions

- 1. Please list any factor(s) you believe have motivated you to volunteer to participate in the Intercultural Peer *e*-learning Global Health project.

- 1. Please comment on your expectations from engaging in intercultural peer learning during your global health project.
