# Supplementary material for: Learning global health: a pilot study of an online collaborative intercultural peer group activity involving medical students in Australia and Indonesia
Source: BMC Med Educ. 2017 Jan 13;17:10. doi: 10.1186/s12909-016-0851-6 (PMC5237179; doi:10.1186/s12909-016-0851-6)
Supplement: Additional file 2: — Post-RIPPLE student survey. (DOCX 15 kb) [file 12909_2016_851_MOESM2_ESM.docx]

**Additional file 2. Post RIPPLE activity student survey**

***Post-RIPPLE activity student survey***

**Intercultural Peer e- Learning in Global Health**

This short survey is intended to explore the effectiveness of intercultural peer learning for teaching global health content. We anticipate this survey will take around five minutes to complete.

***Project design***

1. It was clearly explained why peer learning was used in this part of the Global health course

Strongly disagree disagree agree strongly agree

1. The intended learning outcomes for the peer learning activity were clearly outlined

Strongly disagree disagree agree strongly agree

1. The marking rubric helped me achieve the intended learning outcomes associated with the peer learning activity

Strongly disagree disagree agree strongly agree

1. The use of online tools made it easy to communicate with peers from our overseas partner university

Strongly disagree disagree agree strongly agree

1. The on-campus time allocated to discuss the peer group project using online tools was sufficient

Strongly disagree disagree agree strongly agree

***Peer learning experience***

1. The intercultural peer *e*-learning project gave me an opportunity to learn how overseas students think about global health issues

Strongly disagree disagree agree strongly agree

1. The intercultural group work allowed me to apply and deepen my understanding of global health

Strongly disagree disagree agree strongly agree

1. The intercultural peer project made me aware of similar/different cultural approaches to global health issues

Strongly disagree disagree agree strongly agree

1. Working in small groups helped me achieve the intended learning outcomes of the learning activity

Strongly disagree disagree agree strongly agree

1. I appreciated learning about global health issues by working with my local and international peers, instead of by content delivery via traditional lecture

Strongly disagree disagree agree strongly agree

***Open ended questions:***

- 1. Please comment on how you believe your learning about global health has benefited from your participation in the Intercultural Peer *e*-learning Global Health project?
  2. Please comment on some of the challenges you experienced during your participation in the Intercultural Peer elearning Global Health project.

What aspects and/or components of the Intercultural Peer *e*-learning Global Health project would you change to better facilitate the learning of future student cohorts?
