# Supplementary material for: Learning global health: a pilot study of an online collaborative intercultural peer group activity involving medical students in Australia and Indonesia
Source: BMC Med Educ. 2017 Jan 13;17:10. doi: 10.1186/s12909-016-0851-6 (PMC5237179; doi:10.1186/s12909-016-0851-6)
Supplement: Additional file 3: — List of topics for peer group discussion. (DOCX 16 kb) [file 12909_2016_851_MOESM3_ESM.docx]

**Additional File 3. List of topics for peer group discussion**

| 1. Compare and contrast the epidemiology of dengue in Australia and Indonesia 2. Compare and contrast the treatment of dengue in Australia and Indonesia 3. Compare and contrast the epidemiology and treatment of TB in Australia & Indonesia 4. Compare & contrast the epidemiology of Multi-resistant TB in Australia and Indonesian 5. What methods are used to ensure adherence to TB treatment regimens in Australia & Indonesia 6. Compare the guidelines for malaria prevention for travellers from Indonesia & Australia going to malaria prone areas. 7. What are the trends in ocular trauma in Australian aborigines and rural Indonesia? 8. Compare the common travel health risks if I am visiting NTT (Indonesia) vs NT (Australia) 9. How is climate change affecting tropical infectious diseases in Australia & Indonesia 10. What are the emerging infectious disease challenges of Indonesia & Australia 10. What are the trends in zoonosis in Australia & Indonesia. 11. The definition of the terms: public health, international health, global health & tropical health are debated. What is the dominant contemporary understanding of these terms in Australia & Indonesia. 12. The ‘new’ global health is said to differ from the ‘old’ international health and tropical health by especially placing the care of the individual patient at the same level of priority as prevention for the population Is this true in Australia & Indonesia ? 13. What is he impact of hospital acquired infections on patients and countries when patients seek surgery and other services in distant countries ie medical tourism 14. What is the difference in lab facilities and therefore diagnostic capacity in Kupang (Prof Johannes Hospital) vs Hobart (Royal Hobart Hospital) e.g for infectious diseases 15. How is the assessment and diagnostic probability in the patient with fever different in NTT vs Tasmania? 16. How does the HIV epidemiology and treatment compare between Indonesia & Australia? 17. What is the prevalence of typhoid and any emerging resistant strains in the Asia-Pacific region 18. What are the causes of hospital admitted, childhood diarrhoea in Australia & Indonesia? 19. What is the trends in prevalence of childhood malnutrition & over-nutrition in Indonesia & Australia? 20. Compare and explain the difference of maternal mortality rate (Angka kematian ibu - AKI) and infant mortality rate (angka kematian bayi - AKB) in Australia and Indonesia. 21. Compare and contrast the strategy used to decrease the maternal mortality rate (AKI) and infant mortality rate (AKB) in Australia and Indonesia, especially in NTT and Tasmania. |
| --- |
